# Homoplasy of Retrotransposon Insertions in Toothed Whales

**DOI:** 10.3390/genes14091830

**Published:** 2023-09-21

**Authors:** Liliya Doronina, Lynn Ogoniak, Jürgen Schmitz

**Affiliations:** 1 Institute of Experimental Pathology, ZMBE, University of Münster, 48149 Münster, Germany; l_ogon01@uni-muenster.de; 2 Institute for Evolution and Biodiversity, University of Münster, 48149 Münster, Germany

**Keywords:** retrotransposons, homoplasy, parallel insertions, retrotransposon deletions, whales, Odontoceti, phylogeny of toothed whales

## Abstract

Retrotransposon insertion patterns facilitate a virtually homoplasy-free picture of phylogenetic history. Still, a few most likely random parallel insertions or deletions result in rare cases of homoplasy in primates. The following question arises: how frequent is retrotransposon homoplasy in other phylogenetic clades? Here, we derived genome insertion data of toothed whales to evaluate the extension of homoplasy in a representative laurasiatherian group. Among more than a thousand extracted and aligned retrotransposon loci, we detected 37 cases of precise parallel insertions in species that are separated by over more than 10 million years, a time frame which minimizes the effects of incomplete lineage sorting. We compared the phylogenetic signal of insertions with the flanking sequences of these loci to further exclude potential polymorphic loci derived by incomplete lineage sorting. We found that the phylogenetic signals of retrotransposon insertion patterns exhibiting true homoplasy differ from the signals of their flanking sequences. In toothed whales, precise parallel insertions account for around 0.18–0.29% of insertion cases, which is about 12.5 times the frequency of such insertions among *Alu*s in primates. We also detected five specific deletions of retrotransposons on various lineages of toothed whale evolution, a frequency of 0.003%, which is slightly higher than such occurrences in primates. Overall, the level of retrotransposon homoplasy in toothed whales is still marginal compared to the phylogenetic diagnostic retrotransposon presence/absence signal.

## 1. Introduction

About 50 million years ago (MYA), the terrestrial ancestor of whales and dolphins (Cetacea) moved “back” to life in the water, leaving behind their semi-aquatic sister group of hippopotamuses and the more distant terrestrial artiodactyls, including cows and pigs [1]. In the middle Oligocene (about 36.7 MYA [2]), the cetaceans separated into Mysticeti (baleen whales) and Odontoceti (toothed whales). Toothed whales are small to medium-sized (except for the large sperm whales) and, as the name suggests, are characterized by teeth [3]. Cetaceans diverged profoundly from their land-living relatives to adapt to the aquatic environment, which makes them an attractive group for evolutionary studies. Toothed whales reached public attention with the famous sperm whale Moby-Dick and the television star Flipper, a bottlenose dolphin.

**Figure 1:**
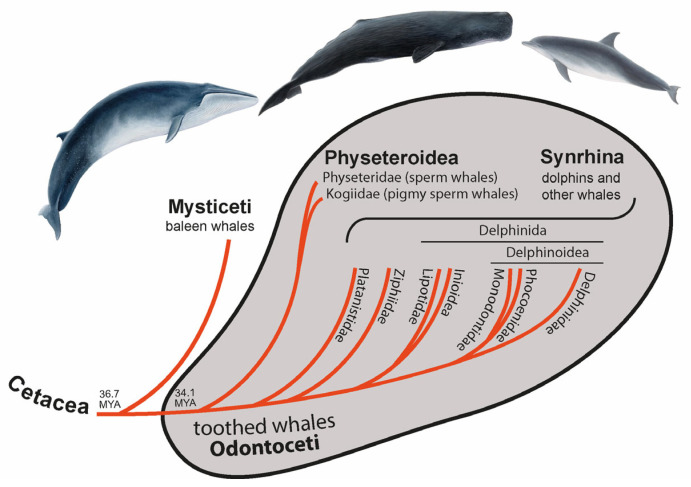
Phylogenetic relationships and terminology of Cetacea. The divergence time of Cetacea and toothed whales was taken from McGowen et al. [2].

Around 2.6 million years (MY) later [2], toothed whales split into Physeteroidea (sperm and pigmy sperm whales) and a clade called Synrhina [4] including Platanistidae (Ganges river dolphins and Indus river dolphins), Ziphiidae (beaked whales), Lipotidae (river dolphins containing the likely extinct baiji), Inioidea (Amazon river dolphins and Franciscana dolphins), Monodontidae (beluga whales and narwals), Phocoenidae (porpoises), and Delphinidae (oceanic dolphins) (Figure 1) [5,6,7]. Around 22.4 MYA Physeteroidea diverged into two lineages—Physeteridae (sperm whales) and Kogiidae (pigmy sperm whales) [2]. Most nuclear data suggest Platanistidae to be the sister group of all other extant Synrhina, followed by Ziphiidae [2,8]. Within Delphinida, the clade Inioidea + Lipotoidea represents a sister group to Delphinoidea. Delphinoidea is divided into two lineages: Delphinidae and Phocoenidae + Monodontidae [2,5,8]. Thus, the toothed whales show well-established higher-level phylogenetic relationships.

Various phylogenetic marker systems offer helpful information about evolutionary relationships, but sometimes present contradicting and, thus, confounding signals. Conflicting markers are most often associated with three primary processes: (1) homoplasy, (2) incomplete lineage sorting (ILS), and (3) hybridization/introgression. Homoplasy represents the independent occurrence of identical phylogenetic signals in two or more lineages (convergence, parallelism) or individual deletions (reversals). The levels of homoplasy differ among the types of markers. For example, homoplasy is higher in sequence data with their low nucleotide change complexity than in retrotransposon presence/absence data with an increased exchange complexity. ILS is the persistence of a polymorphic trait through successive lineage diversifications with subsequent random fixation. Any given marker system may contain signals of ILS depending on speciation frequency and fixation periods. Extremely rapid radiations (illustrated by short phylogenetic branches) may lead to a hard polytomy. Regarding presence/absence data, a hard polytomy is indicated by a nearly equal number of diagnostic markers supporting multiple alternative clustering of lineages (e.g., [9,10]). Hybridization/introgression represents another evolutionary process whereby gene flow occurs among lineages after separation (e.g., [11]). Like ILS, introgression signal frequency depends on the inspected group’s evolutionary history rather than the marker type.

Due to the frequency and primary randomness of their insertions, retrotransposons are especially useful for presence/absence analyses. Retrotransposons are mobile elements that spread in genomes via a copy-and-paste mechanism. A specific retrotransposon in an identical genomic location in two or more lineages generally indicates their common origin. In contrast, its absence in other lineages indicates their more distant relationship. Retrotransposons have been successfully applied to answer many long-standing phylogenetic questions (e.g., [12,13,14]). Due to their virtual homoplasy-free nature [15,16,17], retrotransposons were proposed to be especially suitable for resolving the phylogeny of rapidly radiated groups, where ILS impedes the identification of valid phylogenetic signals.

Short interspersed element (SINE) retrotransposons [18] have been successfully used to investigate cetacean phylogenetic relationships. Shimamura et al. [19] showed the monophyly of cetaceans and their close relationships with *Hippopotamus* and Ruminantia. Nikaido et al. [20,21] found SINE support for the monophyly of Odontoceti, later confirmed in Churakov et al. [22], who also showed that a low level of ILS accompanied the early diversification of cetaceans. Probably due to extensive ILS and/or hybridization, Nikaido et al. [23] found significant conflicts of SINE data in baleen whales. Chen et al. [24] provided a SINE-based reconstruction of Odontoceti phylogeny.

Several studies reported incidental presences of homoplasious retrotransposon signals in Carnivora (e.g., [25,26,27]) and primates [28,29]. However, the only systematic screening of parallel insertions and precise deletions of these phylogenetic markers was performed for *Alu*-SINEs in primates [30]. It revealed a negligibly low level of *Alu*-SINE homoplasy, confirming their virtually homoplasy-free nature.

In the present study, we aimed to identify whether retrotransposon presence/absence patterns have a similarly negligible homoplasy level in a mammalian group more distant to primates, our first target of systematic homoplasy screenings [30], and to determine whether any occurrences of homoplasy compromise the well-established phylogenetic tree. Toothed whales present a viable group for this endeavor because they are distant enough, having been evolving separately from primates for up to 94 MY [31]. They possess moderately varying genomes [32] that enable us to evaluate the precise genomic positions of insertions. Hundreds of thousands of CHR (Cetacea, Hippopotamidae, Ruminantia) SINE elements in whales have been actively inserted over their entire evolutionary history and can be used for homoplasy screenings. Finally, there already exists a reliable phylogenetic tree.

## 2. Materials and Methods

To evaluate the homoplasy of retrotransposons in toothed whales, we screened for their precise parallel insertions and precise deletions. We chose three lineages for the initial screening of precise parallel insertions leading to *Tursiops truncatus* (after the *Platanista minor* split), *Physeter macrocephalus*, and *Kogia breviceps*. The phylogenetic relationship among these three lineages is saddled, and ILS and/or ancestral hybridization are unlikely due to the long period between successive speciations [2]. We screened for retrotransposon insertions in precise genomic locations in the genomes of *T. truncatus*–*Ph. macrocephalus* and *T. truncatus*–*K. breviceps*. To estimate the frequency of precise deletions, we extracted retrotransposons present in all Synrhina except one internal species, indicating an evident loss (deletion) of the element inside this well-supported monophyletic group.

However, our screenings revealed additional conflicting signals in other non-model lineages where we could not clearly distinguish parallel insertions from ILS or ancestral hybridization. Therefore, we performed additional analyses in comparing the retrotransposon presence/absence signals with the signal of their flanking nucleotide sequences (see Section 2.4). 

To evaluate the influence of homoplasious signals on the phylogenetic reconstruction of toothed whales, we performed extensive screenings of conflicting and phylogenetically relevant markers for the toothed whale’s higher phylogenetic relationships. In most comparisons, the high-quality genome of *T. truncatus* (Tur_tru_Illumina_hap.v1) was used to extract a sufficient number of retrotransposon signals.

### 2.1. Inspected Genomes

To begin our search for homoplasious insertions or deletions, we first downloaded the genomes of toothed whales and outgroup species (Supplementary Table S1) and the RepeatMasker reports of *T. truncatus* and *Ph. macrocephalus* from the NCBI (https://www.ncbi.nlm.nih.gov, accessed on 19 September 2023). We used the 2-n-way tool [22] to extract and compare retrotransposon presence/absence patterns after generating the following pairwise whole-genome alignments for the reference genomes *Tursiops* and *Physeter, 1*–7 and 8–13, respectively: (1) *T. truncatus/Pl. minor*; (2) *T. truncatus/Ph. macrocephalus*; (3) *T. truncatus/K. breviceps*; (4) *T. truncatus/Balaenoptera acutorostrata*; (5) *T. truncatus*/*Hippopotamus amphibius*; (6) *T. truncatus*/*Bos taurus*; (7) *T. truncatus*/*Mesoplodon bidens*; (8) *Ph. macrocephalus/K. breviceps*; (9) *Ph. macrocephalus/T. truncatus*; (10) *Ph. macrocephalus/Pl. minor*; (11) *Ph. macrocephalus/B. acutorostrata*; (12) *Ph. macrocephalus*/*H. amphibius*; and (13) *Ph. macrocephalus/B. taurus*. We then compiled the derived two-way alignments in the new n-way project “Whales” (https://retrogenomics.uni-muenster.de/tools/nway/generate, accessed on 19 September 2023).

### 2.2. Transposition-in-Transposition Activity from the RepeatMasker Report

To determine which retrotransposons were active in all the species under consideration, we reconstructed the SINE element activities using Transpositions-in-Transpositions [33] (TinT, https://retrogenomics.uni-muenster.de/tools/tint, accessed on 19 September 2023, Figure 2). TinT calculates the quantity of transposed elements (TEs) that jump in specific other elements: young elements can jump into all other elements, while old elements can only be found in equal or older elements. This provides a relative time scale of element activity to select the right type for specific evolutionary questions. We selected the CHR2 whale SINEs (those covering the entire diversification of toothed whales) for further analyses. From the RepeatMasker reports, we extracted the coordinates of almost full-length SINEs (those missing ≤ 10 nt) with flanking sequences mostly free of other TEs (≥85% of 500-nt left and right flanks free from TEs) using *fastCOEX* (https://retrogenomics.uni-muenster.de/tools/fastCOEX, accessed on 19 September 2023). 

### 2.3. N-Way Analyses

To compare the insertions/deletions in the various alignments, we uploaded the extracted SINE coordinates to n-way [22] and performed screenings, using *T. truncatus* as the reference (standard settings, MUSCLE-based optimization), for the whole-genome pairwise alignments 1–6 (see above). From n-way, we extracted the FASTA files of loci with perfect presence/absence patterns (setting in n-way), in which the genomes of at least two, but not all, cetaceans exhibited the “presence” state, and the outgroup species (*H. amphibius* and *B. taurus*) exhibited an evident “absence”. We extracted parallel insertions, precise deletions, additional conflicting signals, and phylogenetically relevant retrotransposon signals. To receive a more balanced phylogenetic pattern than from the conflict-oriented initial screening, we also performed screenings for underrepresented clades, namely for the Delphinida–Ziphiidae sister group relationship and the Physeteroidea monophyly, using the two-way alignments 1–7 and 8–13 (see above), respectively. Using an NCBI-blast, we manually derived and aligned the retrotransposon signals for additional whale genomes (Supplementary Table S1, Data S1). Retrotransposon insertions of the same SINE type and orientation and identical target site duplications (TSDs) were identified as orthologous insertions. A strict definition of retrotransposon orthology (shift ≤ 3 nt corresponding to the genome position) was taken from [30]. This marginal relaxation from exact insertions was owed to the fact that most retrotransposons are flanked by mutable low-complexity sequences.

N-way extracts targeted insertions of reference species in query genomes together with their flanking sequence regions. In some cases, such flanks contain additional diagnostic insertions, so the number of diagnostic retrotransposons exceeds the number of inspected loci. Such “secondary” insertions are indicated as (b)- or (c)-loci in Supplementary Table S1.

The diversification of toothed whales into sperm whales and the lineages of Synrhina (including dolphins) occurred over ~11.7 (MY) [2], which exceeds the time range over which genomic polymorphism may persist [16]. Therefore, orthologous insertions with the following presence/absence patterns—(1) *T. truncatus* (+), *Ph. macrocephalus* (+), *K. breviceps* (−) and (2) *T. truncatus* (+), *Ph. macrocephalus* (−), *K. breviceps* (+)—were recognized as “precise parallel insertions” if the TSD shift was ≤ 1 nt and as “nearly precise parallel insertions” if the TSD shift was 2–3 nt (corresponding to Doronina et al. [30]). To calculate the frequency of precise parallel insertions, we used the n-way program to estimate the number of the following presence/absence retrotransposon patterns: (1) *T. truncatus* (+), *Pl. minor* (−), *Ph. macrocephalus* (−), *K. breviceps* (−); (2) *Ph. macrocephalus* (+), *K. breviceps* (−), *T. truncatus* (−); and (3) *K. breviceps* (+), *Ph. macrocephalus* (−), *T. truncatus* (−). The orthologous insertions of different retrotransposon types with TSD shifts of 0–1 nt and 2–3 nt were classified as non-homoplasious precise and nearly precise parallel insertions, respectively, as in Doronina et al. [30].

To search for precise deletion cases, we analyzed the insertions occurring in the Synrhina lineage. After supplementing the extracted loci with additional whale genomes (see above, Supplementary Table S1, Data S1), we screened for the cases in which one Synrhina species, after *Pl. minor* diversification, showed an absence state. We assumed that the ancestral state of the retrotransposon in Synrhina was “present” if found in the orthologous loci of *T. truncatus*, *Pl. minor,* and all nested Synrhina lineages except one. In this case, based on the parsimony rule, we proposed this retrotransposon to be precisely deleted from one lineage rather than being parallel-inserted in several synrhinan species multiple times or persistingly polymorphic through all Synrhina speciation with subsequent fixation in all but one (ILS). Since *T. truncatus* was a target species with a predefined “presence, “ we did not screen for precise retrotransposon deletions in its genome. Furthermore, *Pontoporia blainvillei* was excluded from this analysis due to a low assembly quality with many missing loci. To calculate the frequency of precise deletions, we performed an additional n-way screening for *T. truncatus* (+), *Pl. minor* (+).

### 2.4. Comparing Retrotransposon and Flanking Sequence Signals

We compared the phylogenetic signal of the targeted retrotransposons (presence/absence analysis) with the phylogenetic signal of their flanking nucleotide sequences (classical sequence analysis). We expected the phylogenetically relevant retrotransposons to show the same phylogenetic signal as that of the flanking sequences expressing the established toothed whale phylogeny [2,5]. Otherwise, for retrotransposon loci involved in ILS or ancestral hybridization/introgression, the signals of retrotransposons and flanking sequences were expected to be identical but different from the well-supported phylogeny (see mosaic structure of the genome below) [34]. The discordance in signals of retrotransposons and their flanking sequences reflected their different evolutionary histories and indicated true homoplasy.

For orthologous retrotransposon insertions, we also extracted ∼400-nt flanking sequences (~200 nt 5′ and 3′) for species with insertions and the corresponding 400-nt sequences for species with an absence. We constructed concatenated alignments for 17 subsets: 11 subsets for phylogenetically informative loci, four subsets for expected “precise parallel insertion” signals, one subset for supposed ILS-related signals, and one subset for “precise deletions” (Supplementary Table S3a, Data S2). Only subsets containing three or more loci were included in our analyses. Additional varying repetitive elements in flanking sequences were removed from the concatenated alignments. We performed the neighbor-net analyses in SplitsTree [35] with standard settings for net reconstruction and bootstrap analyses for all seventeen concatenated subsets. Furthermore, for the loci from two model subsets of precise parallel insertions—*T. truncatus* [+], *Ph. macrocephalus* [+], *K. breviceps* [−] and *T. truncatus* [+], *K. breviceps* [+], *Ph. macrocephalus* [−]—we extended the flanking regions to ~1000 nt. For this dataset, we performed the neighbor-net analyses locus by locus (Supplementary Table S3b).

### 2.5. Presence/Absence Matrix and Tree Reconstruction

We created a 1/0-matrix for all the identified retrotransposon presence/absence signals (both phylogenetically relevant and conflicting insertions, nexus format, Supplementary Table S2) and used it to generate and analyze phylogenetic trees with MrBayes (MrBayes 3.2 [36]; ctype “irreversible” or “ordered”, mcmc ngen = 20,000, samplefreq = 100, printfreq = 100, and diagnfreq = 1000). PAUP*4.0a [37] was used to derive Dollo parsimony trees using “irrev.up” or “ordered” character transformation in a heuristic search with 1000 bootstrap replicates. The power of the Dollo parsimony was shown in Molloy et al. [38], compared to other variants of parsimony for retrotransposon analyses. We applied “irreversible” and “reversible (ordered)” settings to better evaluate the influence of the homoplasious signals contained in our dataset on the tree reconstruction. We evaluated the statistical significance of our presence/absence data using the KKSC insertion significance test [16].

## 3. Results and Discussion

### 3.1. Extraction of Phylogenetically Relevant and Conflicting Markers

From the RepeatMasker reports of *T. truncatus* and *Ph. macrocephalus,* we extracted 25,327 and 7134 CHR2 SINEs, respectively, for analysis in n-way. Our n-way screenings revealed 1676 perfect presence/absence patterns (clear presence or absence state in n-way for every analyzed species). After in-depth manual analyses of the alignments, we identified 1115 loci containing 1197 orthologous retrotransposon insertions (shift of ≤3 nt, Supplementary Table S1, Data S1).

**Figure 3:**
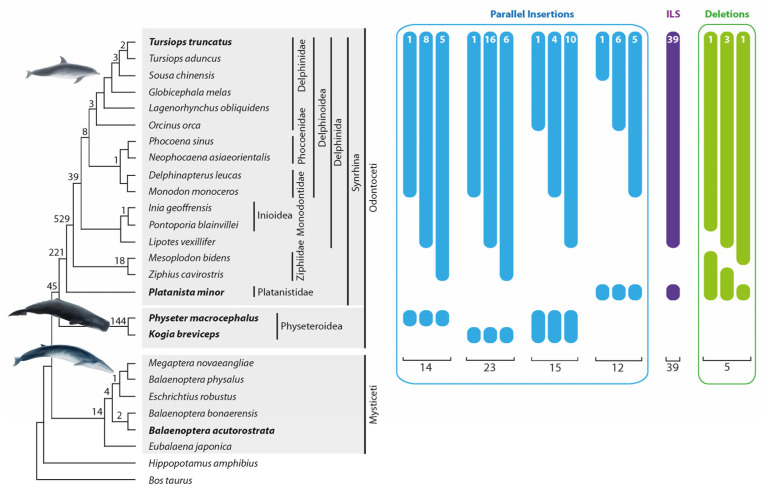
Phylogeny of whales and numbers of precise parallel insertions and deletions. Interrupted bars with numbers represent presence/absence patterns that occurred as a result of precise parallel insertions (blue), incomplete lineage sorting (ILS, purple), or precise deletions (green). The phylogenetic tree topology is taken from McGowen et al. [2]. The bold cetacean species names are those used in n-way screenings to search for homoplasy. Numbers at specific tree nodes are the numbers of phylogenetic diagnostic markers. Fifty-four patterns with nearly precise parallel insertions (2–3 nt shift) and uncertain presence/absence signals are not shown in Figure 3 but are represented in Supplementary Table S1e, Data S1. We used four groups of “parallel insertion” signals (14, 23, 15, and 12 cases) for the homoplasious precise parallel insertion frequency calculation. We detected five deletions in Odontoceti that were used for the deletion frequency estimation.

One thousand thirty-five orthologous insertions supported the established cetacean phylogenetic relationships (Figure 3, the numbers above the tree branches). In the present study, we did not intend to conduct an exhaustive analysis involving sufficient phylogenetic diagnostic markers as were needed to resolve the already well-established cetacean relationships again [2,5]. Nevertheless, we found support for nearly all clades (Figure 3) using the study design described in the Methods section. We received more robust support for some clades than Chen et al. [24]. We detected 45 markers for the Odontoceti monophyly, 221 markers for the monophyly of Synrhina, 529 for the Delphinida and Ziphiidae sister group relationship, and 39 for the Delphinida monophyly (Figure 3). Furthermore, we found the first retrotransposon support for clades previously supported only by other marker systems, including 144 markers for the monophyly of Physeteroidea and 18 for the monophyly of Ziphiidae. We also found 21 phylogenetically informative (Figure 3) and 9 conflicting markers (not shown in Figure 3) in the genome of our outgroup, the baleen whales (Supplementary Table S1a, Data S1 compiles all alignments of all detected retrotransposon loci). The discordant signals are consistent with previous studies describing ILS and ancestral introgression during baleen whale evolution [39,40].

### 3.2. Precise Parallel Retrotransposon Insertions

We detected 14 precise parallel retrotransposon insertions (with a TSD shift ≤ 1 nt) on the lineages leading to *T. truncatus* and *Ph. macrocephalus.* We also found 23 precise parallel insertions and 6 nearly precise insertions (TSD shift 2–3 nt) in the lineages leading to *T. truncatus* and *K. breviceps* (Figure 3, Supplementary Table S1b and Data S1).

Considering that the ancestral lineage of Physeteroidea was maintained for ~11.7 MY without diversification [2], we assume that almost all insertions in the Odontoceti ancestral lineage were fixed before the *Physeter* and *Kogia* lineages separated. Therefore, the retrotransposon insertions with the following presence/absence patterns—*T. truncatus* (+) *Ph. macrocephalus* (+), *K. breviceps* (−) and *T. truncatus* (+) *Ph. macrocephalus* (−), *K. breviceps* (+)—are most probably the result of homoplasy. To verify that the detected insertions were not derived from deep ILS or ancestral hybridization/introgression, we analyzed the nucleotide sequences flanking the precise parallel insertions. Pääbo et al. [41] and Ebersberger et al. [42] showed that some genomes evolved mosaic structures, whereby each genomic locus may have its own evolutionary history and may carry information about phylogenetic affiliations different from that carried by other genomic loci. Within a given recombination unit, the flanking sequences should show the same phylogenetic pattern as that of the retrotransposon insertion, which was demonstrated, for example, for the rapidly radiated laurasiatherian clade [43]. The current neighbor-net analyses of concatenated flanking sequences of 11 phylogenetically relevant groups of retrotransposons also showed identical phylogenetic signals as those of the retrotransposon presence/absence data with strong support (bootstrap > 90%, Supplementary Table S3a). In contrast, the neighbor-net analyses of concatenated flanking sequences for both homoplasious datasets (14 loci with the pattern *T. truncatus* [+], *Ph. macrocephalus* [+], *K. breviceps* [−] and 23 loci with the pattern *T. truncatus* [+], *K. breviceps* [+], *Ph. macrocephalus* [−]) did not show the conflicting retrotransposon patterns but instead revealed strong support for the monophyly of Physeteroidea (bootstrap 100%, Supplementary Table S3a). To check whether the heterogeneity of loci in concatenated datasets could drive these results, we extended the flanking regions of retrotransposons up to ~1000 nt in the *T. truncatus* (+) *Ph. macrocephalus* (+), *K. breviceps* (−) and *T. truncatus* (+) *Ph. macrocephalus* (−), *K. breviceps* (+) datasets, and performed locus by locus neighbor-net analyses in SplitsTree [35]. All 14 loci from the *T. truncatus* (+) *Ph. macrocephalus* (+), *K. breviceps* (−) dataset and 22 of the 23 loci from the *T. truncatus* (+) *Ph. macrocephalus* (−), *K. breviceps* (+) dataset provided substantial support for the *Physeter*–*Kogia* monophyly (all 36 loci with bootstrap > 80%, and 31 of them with bootstrap > 90%, Supplementary Table S3b). These results strengthen our initial assumption that such conflicting retrotransposon insertions were neither inherited from a common ancestor nor occurred via hybridization but instead represent true homoplasy.

We also collected additional data illustrating precise parallel insertions in cetaceans. In 19 conflicting cases (15 loci with 0–1 nt SINE shifts [Figure 3] and four loci with 2–3 nt shifts; Supplementary Table S1b, Data S1), SINE presence/absence patterns suggested the stringent affinity of the Physeteroidea lineage to Delphinida or their different subgroups. The neighbor-net analyses of the concatenated flanking sequences represented a strong support for the traditional Synrhina clade with the exclusion of Physeteroidea (bootstrap 92.5%, Supplementary Table S3a). The identified conflicting SINE signals were probably the result of precise parallel insertions into dolphins and the ancestral Physeteroidea lineage. Furthermore, we found 15 retrotransposon signals (12 loci with 0–1 nt SINE shifts [Figure 3] and three loci with 2–3 nt shifts, Supplementary Table S1b, Data S1) present in some *T. truncatus*-related cetaceans and *Pl. minor*. The analysis of concatenated flanking sequences supported the traditional phylogeny of the toothed whales, suggesting the detected retrotransposon presence/absence patterns, to be precise parallel SINE insertions.

### 3.3. Frequency of Parallel Insertion Homoplasy

To calculate the frequency of homoplasious precise parallel insertions, we performed n-way screenings to identify the total number of retrotransposons inserted in the lineage leading to *T. truncatus* (after *Pl. minor* diverged) and in the lineages leading to *Ph. macrocephalus* and *K. breviceps* after they split. The n-way screenings for *T. truncatus* (+), *Pl. minor* (−), *Ph. macrocephalus* (−), *K. breviceps* (−) and for *Ph. macrocephalus* (+), *K. breviceps* (−), *T. truncatus* (−) revealed 7782 and 36 autapomorphic CHR2 SINE insertions in *T. truncatus* and *Ph. macrocephalus*, respectively. We estimated the frequency of parallel insertions in the *T. truncatus* and *Ph. macrocephalus* lineages to be 0.18% (14/(7782 + 36 + 14) × 100%) (see [30]). The n-way screening for *K. breviceps* (+), *Ph. macrocephalus* (−), *T. truncatus* (−) revealed 94 autapomorphic CHR2 SINE insertions in *K. breviceps*. We estimated the frequency of parallel insertions in the *T. truncatus* and *K. breviceps* lineages to be 0.29% (23/(7782 + 94 + 23) × 100%).

The analysis of two *Ph. macrocephalus* (ASM283717v5, Physeter_macrocephalus-2.0.2) and two *K. breviceps* (mKogBre1_haplotype_2, KogBre_v1_BIUU) genomes available in the NCBI revealed no retrotransposon polymorphisms in *Ph. macrocephalus* for the *T. truncatus* (+), *Ph. macrocephalus* (+), *K. breviceps* (−) retrotransposon patterns. However, six of 23 retrotransposons inserted parallelly in *T. truncatus* (+) and *K. breviceps* (+) remain polymorphic in the *K. breviceps* species. The recent activity of the CHR2 SINEs in the *K. breviceps* lineage and the slightly increased number of CHR2 SINEs in the *K. breviceps* genomes (9447 and 7134 complete CHR2s in retrotransposon-free genomic regions in *K. breviceps* and *Ph. macrocephalus*, respectively) possibly explain the increased number of precise parallel insertions in the pair *T. truncatus*–*K. breviceps* compared to *T. truncatus*–*Ph. macrocephalus*.

To evaluate the frequency of additionally found parallel insertions in *T. truncatus* and the entire Physeteroidea lineage, we performed an n-way screening for two patterns—(1) *T. truncatus* (+), *M. bidens* (−), *Pl. minor* (−), *Ph. macrocephalus* (−), *K. breviceps* (−), and (2) *Ph. macrocephalus* (+), *K. breviceps* (+), *T. truncatus* (−)—that revealed 5403 and 234 loci, respectively. We estimated the frequency of parallel insertions to be 0.27% (15/(5403 + 234 + 15) × 100%).

We also performed an additional n-way screening for the (1) *T. truncatus* (+), *M. bidens* (−), *Pl. minor* (−) and (2) *Pl. minor* (+), *T. truncatus* (−), *M. bidens* (−) patterns, and estimated the frequency of precise parallel insertions in the *T. truncatus*-related cetaceans and *Pl. minor* to be 0.18% (12/(6616 + 143 + 12) × 100%). Thus, our additional analyses of parallel insertions confirmed that homoplasious retrotransposon insertions in toothed whales occur with a frequency of 0.18–0.29%.

An analysis of different primate lineages revealed 2–6 parallel insertions per lineage pair with a parallel insertion frequency of 0.01–0.04% [30]. Thus, the present study indicates that toothed whales exhibit a frequency of parallel insertions varying from 7 to 18 times that in primates. This cannot be explained by a longer period of evolution or speciation in toothed whales because it was intermediate to that in the human–chimpanzee–rhesus macaque and human–bushbaby–lemur model systems. One potential reason for the difference between primates and whales may be the reduced genome size in cetaceans (2.4–2.6 Gb [44]) compared to the larger genomes in primates (~3.2 Gb in our reference primate group; see [30]), which was evoked mainly by retrotransposon insertions over the last 50 MY [45]. The cetaceans’ comparatively smaller genomes might have forced repeated independent insertions into fewer suitable genomic insertion sites. It has been shown that retrotransposon insertion sites are not entirely randomly selected [46]. For example, LINE1 elements tend to integrate into (5′) TT/AAAA genomic motifs in humans and rodents [47,48]. It should be noted that SINEs are non-autonomous retrotransposons that co-mobilize via active autonomous LINE1 elements using their reverse transcriptase and endonuclease [49], thus sharing their site preferences. However, the LINE1 target site preference has not explicitly been investigated in whales. A slightly different target site preference of the whale SINE-associated autonomous LINE1 elements might also have contributed to a higher level of homoplasy in toothed whales.

### 3.4. Non-Homoplasious Retrotransposon Parallel Insertions

We detected 35 non-homoplasious independent parallel insertions in cetaceans in two lineages at the same locus (Supplementary Table S4). Twenty were inserted precisely (≤1 nt shift) in the *T. truncatus*-related lineage and baleen whales, and seven in the *T. truncatus*-related clade and Physeteroidea. In 26 of the 27 cases, the SINE types in the two cetacean lineages could be clearly distinguished, and, in one case, the SINEs were also inserted in the opposite orientations. In the remaining one case, we identified CHR2B_Ttr SINEs in both lineages. However, there was a significant difference in the sequence patterns of SINE tails in the two lineages. Therefore, we also categorized these cases as non-homoplasious precise insertions. The other eight detected non-homoplasious cases included nearly precise insertions (four loci with 2–3 nt shift) or precise insertions in the *T. truncatus*-unrelated lineages (four loci).

The number of non-homoplasious precise insertions in cetaceans is much larger than in the human–rhesus macaque or chimpanzee–rhesus macaque lineage pairs [30]. It is not surprising, taking into account the fact that there were only ~7.5 MY after the human–chimpanzee diversification [50] for the human lineage to accumulate the SINE insertions in the same genomic loci as in the rhesus macaque lineage. A comparable number of independent insertions was found in the human–bushbaby lineages; however, the diversification of human–bushbaby–lemurs lasted twice as long as that of cetaceans. Therefore, as for homoplasious insertions, the number of non-homoplasious precise insertions in whales is higher than we expected from the primate data.

### 3.5. ILS in Toothed Whales

Interestingly, we found solid, conflicting signals (39 loci) for the Delphinida-Platanistidae group affiliation (Figure 3, Supplementary Table S1c, Data S1). Like other conflicting signal groups, neighbor-net analysis of the concatenated flanking sequences contradicted the retrotransposon pattern and revealed the phylogenetically relevant Delphinida + Ziphiidae grouping. However, the bootstrap support was weak (bootstrap 66.1%, Supplementary Table S3a). Separate analyses of the flanking sequences of each locus revealed that around one-third of all loci supported the same phylogeny as the retrotransposons (Delphinida + Platanistidae). Our results suggest that the Delphinida– Platanistidae dataset contains homoplasious insertions and ILS signals. ILS accompanies rapidly radiating clades, indicating that the time between diversifications is shorter than the time required to fix genomic polymorphisms in the population. The diversification of ancestral cetaceans into Delphinida, Ziphiidae, and Platanistidae occurred in less than one MY [5]. Previously, it was shown that, in several mammalian clades, ILS accompanied the diversifications that occurred over 2 MY [16,27]. Likewise, the laurasiatherian diversification into four lineages over less than 2 MY led to such a strong level of ILS that there is still a lack of consensus concerning the correct phylogeny of these species [51]. The phylogenetic relationships of the Delphinida–Ziphiidae–Platanistidae lineages were controversial according to mitochondrial sequence data [52,53]. However, nuclear sequence analyses revealed surprising congruent tree topologies supporting a Delphinida–Ziphiidae sister group relationship [2,8]. We also found an overwhelming and significantly relevant signal for the Delphinida–Ziphiidae affiliation (529:39 markers, one-directional KKSC insertion significance test *p* < 3.8 × 10^−111^). The relatively low level of ILS suggests a tiny population size that enabled the quick fixation of the retrotransposon polymorphisms and the accumulation of phylogenetically solid signals in this group. Assuming a medium generation time of 21.1 years [54] and an ancestral effective population size of 13,000 [55], the fixation of markers (generation time t = 4*N_e_*) in the ancestral *T. truncatus* population was about one MY, which is only two times faster than in primates. However, the ancestral toothed whale population might have gone through a local bottleneck around 33–32 MYA, associated with, for example, the major oceanic restructuring that occurred during this period [5].

### 3.6. Precise Retrotransposon Deletions

Screening for precise deletions in toothed whales focused on the *T. truncates*–*Pl. minor* monophyletic group. Within this group, we searched for retrotransposons present in all analyzed cetaceans except for individual nested species. Thus, we had equal chances of finding precise deletions in any of the 13 analyzed species that diversified from the *T. truncatus* ancestral lineage after *Pl. minor* diverged (*T. truncatus* and *P. blainvillei* were not included, see Section 2). We detected five cases of precise deletions within the *T. truncatus*–*Pl. minor* group: one in *Lipotes vexillifer*, one in *Ziphius cavirostris*, and three in *M. bidens* (Figure 3, Supplementary Table S1d, Data S1). Considering that we searched for precise deletions in 13 lineages, we estimated the average precise deletion rate to be 0.38 (5/13) per lineage. The n-way screening for the *T. truncatus* (+), *Pl. minor* (+) retrotransposons revealed 11,164 insertions that comprised the substrate for precise deletions in the analyzed group. We estimated the frequency of precise deletions in toothed whales to be 0.003% (0.38/(11,164 + 5) × 100%) (see [30]). The total number of precise deletions found in primates [30] was slightly higher than in toothed whales (12 vs. 5). However, primate genomes contained significantly more *Alu* SINE elements that comprised the substrate for retrotransposon deletions (54,037 *Alu*s vs. 11,169 CHR2 SINEs). Thus, the estimated frequency of retrotransposon precise deletions in whales is slightly higher than in primates. It should be noted that all precise deletion events found in primates occurred over ~7.5 MYs (after human–chimpanzee diversification [50]). In contrast, the analyzed toothed whales had ~32 MYs to lose their retrotransposons.

### 3.7. Retrotransposon-Based Phylogenetic Tree Reconstruction

Among the 1197 extracted retrotransposon presence/absence patterns, we detected 1035 phylogenetically relevant markers, 77 precise and nearly precise parallel insertions, 5 precise deletions, and 39 ILS-related signals. The remaining 41 loci contained the following: single, random cases of conflicting signals in cetacean lineages out of our focus; cases for which parallel insertions and precise deletions could not be distinguished; or cases with complex evolutionary scenarios in which possibly more than one homoplasious event had occurred (Supplementary Table S1, Data S1). We reconstructed the Bayesian and PAUP phylogenetic trees (irreversible and ordered character transformation) using all the detected SINE signals, including the conflicting ones. Despite the discordant SINE patterns resulting from homoplasy and ILS, our phylogenetic reconstructions of toothed whale higher-level relationships largely agree with previous cetacean phylogenetic reconstructions ([2,5]; Supplementary Figure S1a,b). The reconstructed topology of baleen whales differs from the published ones [2,39,40]. It should be noted that the baleen whale phylogeny was out of our focus, and our dataset contains only a few occasionally found markers, which are not sufficient for reconstructing a representative tree topology.

Thus, the frequency of precise parallel insertions and deletions of cetacean SINEs was higher than in primates. However, the general level of homoplasy is still low and does not compromise the use of retrotransposon insertions as highly reliable phylogenetic markers.

## 4. Conclusions

Even though the homoplasy rate in toothed whales is 7–18 times higher than in primates, the overall level is still low. Retrotransposon presence/absence data can still be considered virtually homoplasy-free. The drastic change in cetacean life as they made their way back into water might have influenced the level of homoplasy, so it would be interesting to analyze the frequency of homoplasy in another distant phylogenetic group that experienced a more linear evolutionary history in water or on earth.

## Figures and Tables

**Figure 2:**
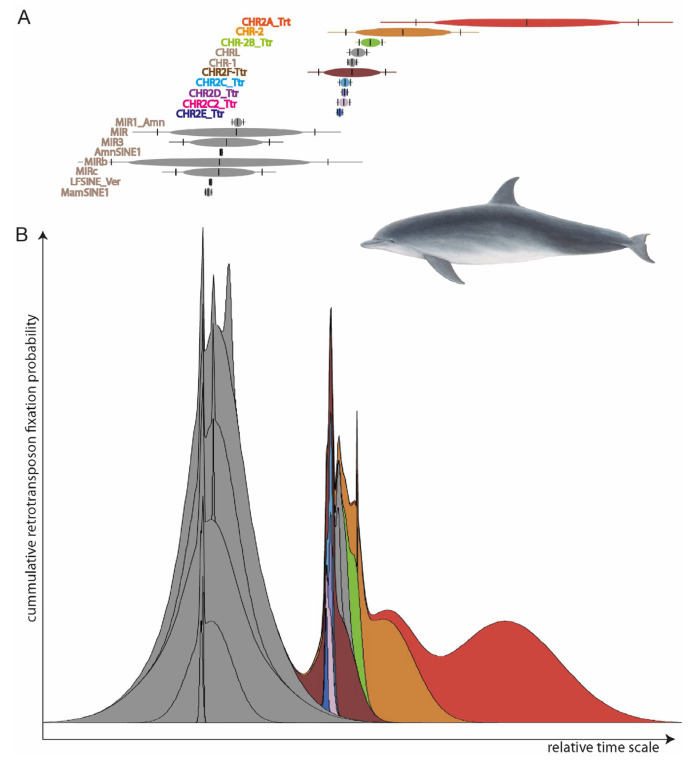
TinT patterns of element activities for (**A**) individual SINE elements and (**B**) cumulative TinT. Two major SINE activity waves are visible: one in the common ancestor of mammals (grey MIR-related SINEs active before the main mammalian speciation), and one that probably correlates to the transition to water 50 MYA (multi-colored CHR SINEs).

## Data Availability

All retrotransposon and sequence data are present in the Supplementary Materials.

## Supplementary Materials

The following supporting information can be downloaded at: https://www.mdpi.com/article/10.3390/genes14091830/s1, Table S1: Presence/absence table for all diagnostic retrotransposon insertions; Table S2: 1/0 matrix of all diagnostic retrotransposons; Table S3: Neighbor-net analyses and bootstraps of flanking regions; Table S4: Presence/absence table for non-homoplasious precise retrotransposon insertions; Data S1: Alignments of all detected retrotransposon loci; Data S2: Concatenated retrotransposon flanking sequences; Figure S1a,b: Bayesian and PAUP phylogenetic trees of the 1/0 retrotransposon matrix.
