# Supplementary figures and images for: Homoplasy of Retrotransposon Insertions in Toothed Whales
Source: Genes (Basel). 2023 Sep 21;14(9):1830. doi: 10.3390/genes14091830 (PMC10531181; doi:10.3390/genes14091830)

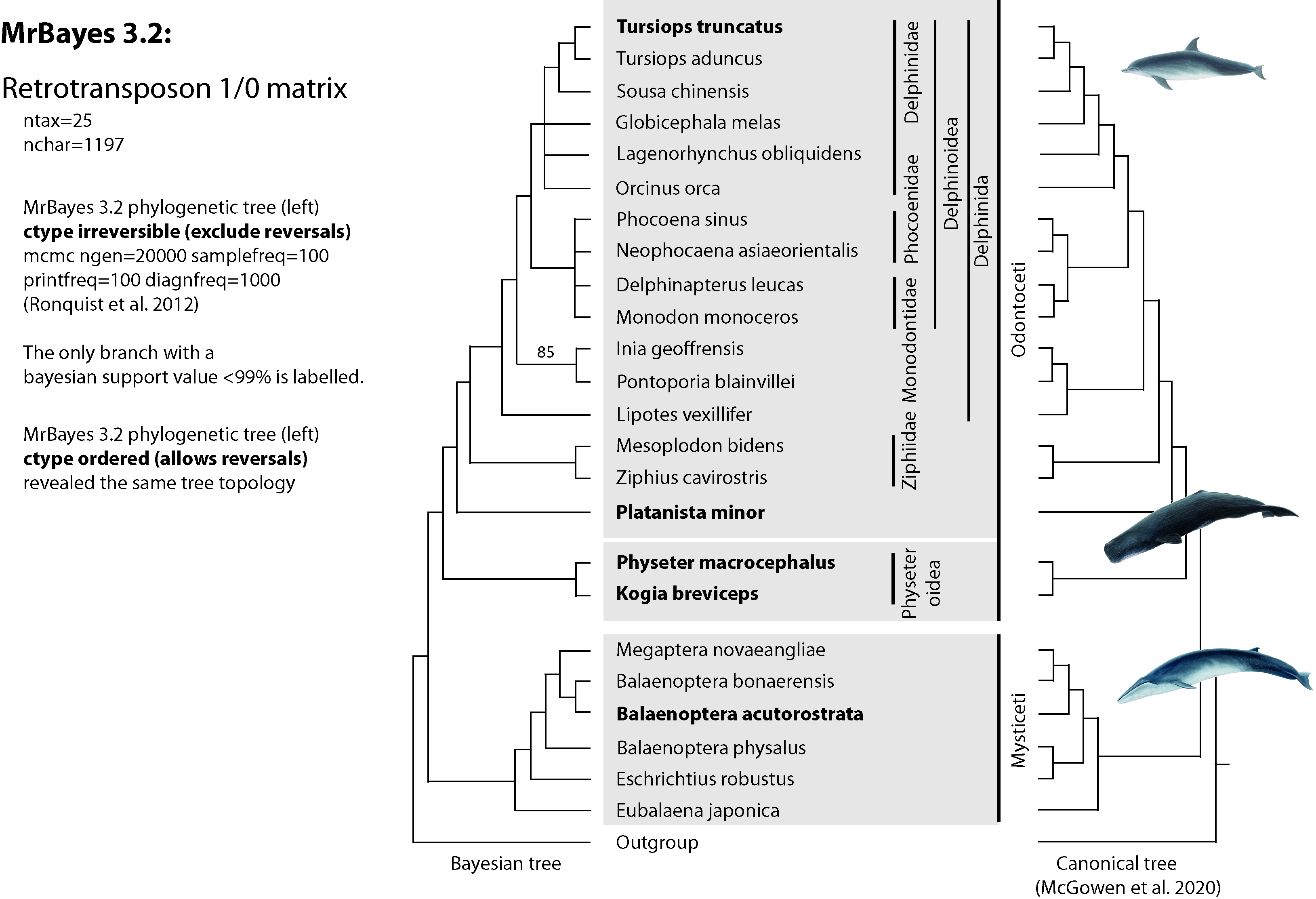

Supplement: Supplementary file 1 [file genes-14-01830-s001.zip › Supplementary_Figure_S1a.jpg]

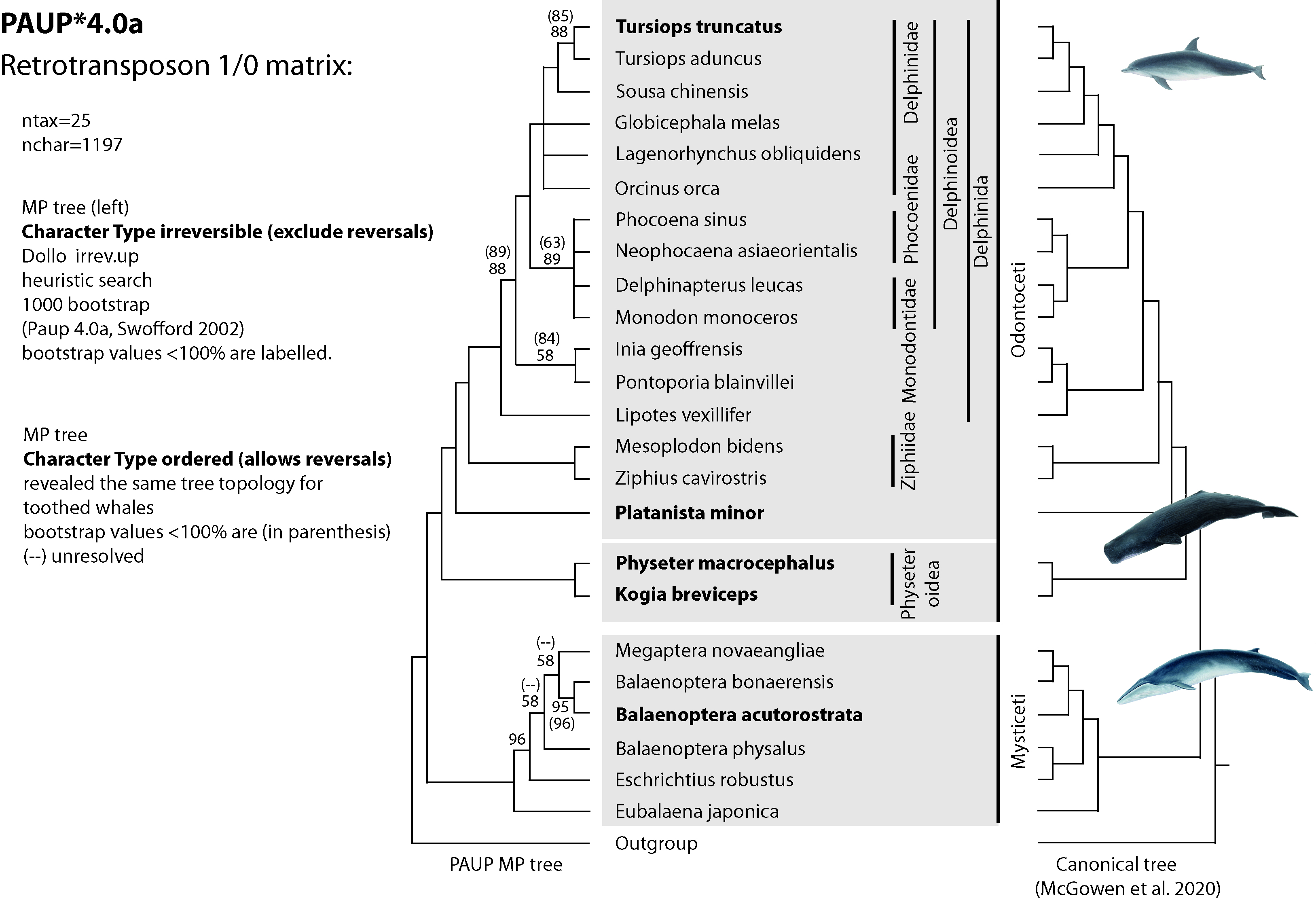

Supplement: Supplementary file 1 [file genes-14-01830-s001.zip › Supplementary_Figure_S1b.jpg]
